# Secretory Autophagy and Its Relevance in Metabolic and Degenerative Disease

**DOI:** 10.3389/fendo.2020.00266

**Published:** 2020-05-05

**Authors:** Claudio Daniel Gonzalez, Roxana Resnik, Maria Ines Vaccaro

**Affiliations:** ^1^Department of Pathophysiology, Institute of Biochemistry and Molecular Medicine (UBA-CONICET), School of Pharmacy and Biochemistry, University of Buenos Aires, Buenos Aires, Argentina; ^2^CEMIC University Institute, Buenos Aires, Argentina

**Keywords:** unconventional protein secretion, IL-1β, aggregate-prone proteins, macroautophagy, ATG (autophagy-related) proteins

## Abstract

Proteins to be secreted through so-called “conventional mechanisms” are characterized by the presence of an N-terminal peptide that is a leader or signal peptide, needed for access to the endoplasmic reticulum and the Golgi apparatus for further secretion. However, some relevant cytosolic proteins lack of this signal peptides and should be secreted by different unconventional or “non-canonical” processes. One form of this unconventional secretion was named secretory autophagy (SA) because it is specifically associated with the autophagy pathway. It is defined by ATG proteins that regulate the biogenesis of the autophagosome, its representative organelle. The canonical macroautophagy involves the fusion of the autophagosomes with lysosomes for content degradation, whereas the SA pathway bypasses this degradative process to allow the secretion. ATG5, as well as other factors involved in autophagy such as BCN1, are also activated as part of the secretory pathway. SA has been recognized as a new mechanism that is becoming of increasing relevance to explain the unconventional secretion of a series of cytosolic proteins that have critical biological importance. Also, SA may play a role in the release of aggregation-prone protein since it has been related to the autophagosome biogenesis machinery. SA requires the autophagic pathway and both, secretory autophagy and canonical degradative autophagy are at the same time, integrated and highly regulated processes that interact in ultimate cross-talking molecular mechanisms. The potential implications of alterations in SA, its cargos, pathways, and regulation in human diseases such as metabolic/aging pathological processes are predictable. Further research of SA as potential target of therapeutic intervention is deserved.

## Autophagy

Autophagy is an evolutionarily conserved cellular process induced by nutrient starvation or lack of growth factors that sequester and delivers cytoplasmic components to the lysosome for degradation (1). The classical functions of autophagy are nutrient recycling functions by bulk sequestration from the cytoplasm (2). It is also involved in the cytoplasmic component quality control by removing specifically damaged or aging organelles, such as depolarized mitochondria (3). Autophagy is also important in proteostasis, sequestering and degrading long-life proteins and invading microbes (4). However, recent reports found that autophagy also presents non-canonical functions (5), especially regulating unconventional secretory processes. Thus, a novel non-degradative role of autophagy has emerged, raising the concept of Secretory Autophagy (SA) (6–8).

In the classical view, according to the pathway that cargo follows to reach the lysosomal compartment, there are three mayor types of canonical degradative autophagy. These types are: microautophagy/endosomal microautophagy (9, 10), chaperone-mediated autophagy (CMA) (11, 12), and macroautophagy. The last one is characterized by the engulfment of cytoplasmic contents by a double membrane vesicle, named autophagosome. Therefore, macroautophagy (hereafter mentioned as autophagy) is distinguished by the formation of the autophagosome as its characteristic and representative intracellular organelle (13).

**Figure 1:**
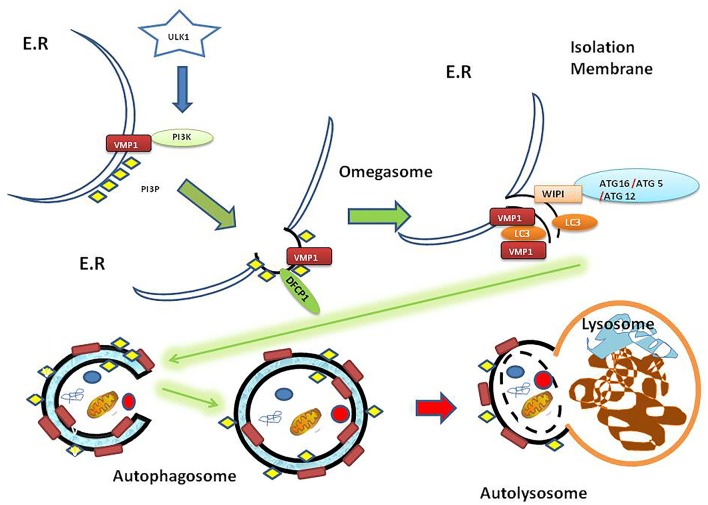
Autophagy overview diagram flow. Autophagosome biogenesis is mediated by ULK1 activation. Here is shown that VMP1, a transmembrane protein, recruits PI3K complex on the ER surface. Then DFCP1 recognizes the PI3P subdomain on the omegasome structure. Besides, WIPI proteins recruit the ATG16-ATG5-ATG12 protein complex on the isolation membrane. In turn, the ATG16-ATG5-ATG12 complex mentioned above mediates LC3 lipidation on the membrane. The genesis of the autophagosome as a double membrane vesicle allows carrying its cargo to the lysosome where the cargo is eventually degraded in the resulting autolysosome as a final structure [reviewed in (15)]. ER, endoplasmic reticulum; PI3K, phosphatidylinositol 3-kinase; PI3P, phosphatidylinositol (3,4,5) triphosphate (PI3P); ULK1, Unc-51-like kinase 1; VMP1, Vacuole Membrane Protein 1; DFCP1, Double FYVE-containing protein 1 (omegasome marker); WIPI, WD40-repeat phosphoinositide-interacting protein (isolation membrane marker); LC3, Microtubule-associated proteins 1A/1B light chain 3B (vesicle maturation/cargo recognition); ATG12, Autophagy-related protein 12 (member of ATG12-ATG5-ATG16L involved in LC3 conjugation to autophagosome membrane); ATG5, autophagy-related protein 5; ATG16, autophagy-related protein 16.

The autophagic process involves the fusion of the outer membrane of the autophagosomes with lysosomes to deliver the inner vesicle with its cargo to the degradation compartment forming the autolysosome. In the autolysosome, the inner vesicle is degraded, and its products recycled. So far, more than 100 molecules have been related to autophagy regulation and were named ATG molecules (14). Two signaling pathways are associated with autophagy induction: those involve mTOR and AMPK activation. These signaling pathways can sense the environmental, nutritional and energetic status of the cell and promote autophagy through the ULK1-complex, which is the first member of the core molecular machinery in the autophagosome biogenesis [reviewed in (15, 16)]. In brief: Following ULK1 complex activation, the transmembrane protein VMP1 (17) recruits on the ER surface contact site (18) where the first structure in the autophagosome biogenesis, called omegasome, is formed. VMP1 also interacts with the BH3 domain of BECN1 recruiting the kinase complex PI3KC3-C1 to the autophagosome membrane (19). The events that lead to the initial structures (isolation membrane) are followed by the BECN1-PI3K complex activity that phosphorylates the autophagosome membrane and two ubiquitin-like systems ATG12 and LC3 that promote the proper recognition of PI3P (20). In this way, cytoplasmic ATG12 is covalently attached to a C-terminal glycine of ATG5. Furthermore, the ATG5-ATG12 complex promotes LC3 conjugation to phosphatidylethanolamine (PE) on the autophagosomal membrane and this process is mediated by ATG16L, which interacts with ATG5 to eventually form the ATG12-ATG5-ATG16L complex [(21); Figure 1].

It is well-known that LC3 plays a central role in autophagy being involved in vesicle elongation, maturation, fusion of autophagosome-lysosome, and even as an adaptor to cargo recognition (22, 23). The lipidated LC3, (LC3B) is present at the isolation membrane and in the autophagosome, in both sides of the membrane. The arrival of autophagosome to the lysosome, is a fusion dependent mechanism of the HOPS complex, through STX17 (24), and RAB7 (25). Thus, LC3 from the inner membrane of the autophagosome is degraded with the cargo. LC3 localized in the external membrane is cleaved from the PE by ATG4B and then recycled (26–28).

Apart from its physiological/homeostatic function, autophagy is also considered as a cell adaptation-to-stress process, which frequently starts as a consequence of organelle damage caused by oxidative species and other stress conditions. Any specific sequestration of a selected type of cargo by autophagy for its delivery to the lysosome is called *selective autophagy* (29). Selective autophagy has a role in intracellular homeostasis, mediating the specific degradation of cytoplasmic material such as aggregated proteins or damaged mitochondria (30). Interactions between autophagy receptors and ubiquitin-like proteins constitute the molecular basis of selective autophagy. In selective autophagy, a cargo-receptor-protein, such as p62, makes the connection between the selected cargo and LC3 in the autophagosomal membrane (31). Importantly, selective degradative autophagy is involved in the cellular response to complex diseases, such as metabolic/aging pathological processes, by the specific degradation of aggregation-prone or aggregated proteins (30, 32) and organelles. These well-studied aspects of degradative autophagy are widely considered an attractive target for therapeutic strategies (33).

## Secretory Autophagy

In most cases, especially in exocrine glands and neurons, proteins are secreted by exocytosis (34). The amino-terminal signal peptide (leader sequence) leads eukaryotic secretory proteins into the endoplasmic reticulum (ER), following a well-defined secretory pathway via the Golgi apparatus and eventually progress to the cell surface through vesicular flow. However, some relevant cytosolic proteins lack of this signal peptides and are not able to enter the endoplasmic reticulum (ER). Therefore, they should be secreted by different unconventional or “non-canonical” processes that differ from the classical ER-Golgi pathway (35–37). The autophagy machinery participates in at least one of these pathways. Thus, as mentioned above, this autophagy-dependent secretion pathway is also referred to as SA (6–8).

SA is becoming of increasing relevance to explain the secretion of a series of peptides that have critical biological importance. Interestingly, SA has been shown to play a role in the release of aggregation-prone proteins. This highlights the pathophysiological relevance of this novel, but still not fully elucidated autophagy mediated secretory pathway (38, 39). Autophagy has been also involved in extracellular export of cytosolic organelles, such as mitochondria that can also be released by secretory autophagy (40). Furthermore, different types of non-canonical autophagy have been involved in pathogen released from infected cells (41) and associated with the unconventionally trafficking of proteins to the plasma membrane (42).

Interleukin-1β (IL-1β) secretion is mediated by SA. LC3B-positive carrier sequesters IL1β from the cytosol and fuses with the plasma membrane to release this cytokine through a SA process (6–8, 43). IL-1β release seems to request the participation of the TRIM family proteins as receptors for cargo to be secreted. It has been reported that the TRIM family interacting with SEC22B, as well as some Qa-SNARE (syntaxins 3 and 4), and Qbc SNARE (SNAP 23 and 29) are needed to promote the secretory release of IL-1β. Also, these molecules are needed for other unconventional secretion processes, such as those involving Lysozymes, Cathepsin A, B, C, S, Z, and other dipeptidyl-peptidases and Tubulin (7, 44). Other SA cargos that do not contain a signal peptide are IL-18 and HMGB1 (45, 46). SA is not restricted to inflammasome substrates and autophagic mediated secretion of other cytosolic proteins lacking leader peptide have been reported, such as Galectin-3, Ferritin, and Annexin-I (47).

It has been observed that SA is involved in α-Synuclein aggregates associated with Parkinson's disease (48–50). SA has been linked to the release of aggregates of amyloid-beta (Aβ) peptide associated with Alzheimer's disease (46). A decrease in Aβ secretion and extracellular Aβ plaque formation and an increase of intracellular Aβ aggregate in the perinuclear zone of neurons were reported in neuron-specific ATG7-deficient mice. Also, ATG7 was able to rescue the ability to release Aβ, whereas the induction of autophagy with rapamycin decreased the secretion of Aβ from wild-type primary neurons (46). Moreover, the secretion of Parkinson's disease- and cancer-associated protein Park7/DJ-1 is mediated by SA. Park7 secretion is induced by autophagy through AMPK and ULK1 activation and it is suppressed in Atg5, Atg9, or Atg16lL deficiency animals (51). On the other hand, the inhibition of degradative autophagy was reported to induce the unconventional secretion of α-Synuclein and Huntingtin protein (50). Therefore, growing evidence accumulates to point at a relevant role of SA in pathological protein aggregate secretion and their intracellular accumulation as mechanisms of cell response to degenerative diseases.

SA has a role in intestinal defense mechanisms being involved in the secretion of Lysozyme in Paneth cells, mediated by ATG161L (52). SA is triggered by bacteria-induced ER stress and it is disrupted in Paneth cells of mice harboring ATG16L1, which is a variant of ATG16L related to high risk for Crohn's disease. Another example is the secretion of CFTR (cystic fibrosis transmembrane conductance regulator): knockdown of ATG5 and ATG7, and treatment with autophagy inhibitors, such as wortmannin and 3-methyladenine, abolished the unconventional secretion of CFTR stimulated by ER stress and an ER-to-Golgi blockade (53).

Several ATG-proteins involved in the biogenesis of the autophagosome have been directly related to the molecular mechanisms in SA (Table 1). However, the participation of the autophagosomes in the membrane trafficking pathways of SA is not completely elucidated (37, 58). The analysis of the mechanisms underlying unconventional protein secretion includes a wide range of pathways that goes from different forms of plasma membrane translocation to the generation of extracellular vesicles (EVs).

**Table 1:** Examples of autophagy related molecules and modulators affecting unconventional protein secretion.

**Autophagy-related molecules and modulators**	**References**	**Protein secretion**	**Potentially affected disease**
ULK1-complex	(51)	Park7	Parkinson's disease
ATG5	(8, 51, 53–55)	IL-1β; Park7; CFTR; IDE	Parkinson's disease; Cystic Fibrosis; Alzheimer's disease; several chronic inflammatory diseases^*^
ATG16L1	(51, 52)	Lysozyme; Park7	Parkinson's disease; Crohn's disease
ATG7	(50, 53, 55–57)	α-synuclein; CFTR; Amyloid beta; IDE	Parkinson's disease; type 2 diabetes; cystic fibrosis; Alzheimer's disease
LC3	(8)	IL-1β	Several chronic inflammatory diseases^*^; carcinogenesis
BECN1	(43, 55)	IL-1β; IDE	Alzheimer's disease
SEC22B	(7)	IL-1β	Several chronic inflammatory diseases^*^; carcinogenesis
TRIM16	(7)	IL-1β	Several chronic inflammatory diseases^*^; carcinogenesis
3-MA	(8, 53)	IL-1β; CFTR; IDE	Cystic fibrosis; Alzheimer's disease
Bafilomycin A	(45, 55)	IDE; IL-1β	Alzheimer's disease
Rapamycin	(46)	Amyloid beta	Alzheimer's disease
Spautin 1	(57)	Amyloid beta	Alzheimer's disease
Starvation	(8, 54)	IL-1β	Several chronic inflammatory diseases^*^; carcinogenesis

* *Including: rheumatoid arthritis, inflammatory bowel disease; autoimmune thyroiditis; type 2 diabetes*.

EVs are a heterogeneous group of cell-derived membranous structures that can be originated from the endosomal system and micro-vesicles that are shed from the plasma membrane (59). A type of EVs with a diameter range between 40 and 120 nm were called exosomes. It is interesting to highlight that the secretion of exosomes was also related to the SA pathway (60). This finding suggests that exosomes can be another cargo for SA. Moreover, some cytoplasmic components, from single molecules to organelles, could be involved in the SA mechanism and may also work as SA cargos (61). However, the intracellular membrane trafficking involved in the exosome secretion pathway largely remains poorly understood. In the classic view, exosome biogenesis would start when early endosomes mature into late endosomes or MVBs (Multi Vesicle Bodies) that fuse with the plasma membrane to release the extracellular vesicles to the environment (62). Hence, during this process, the endosomal membrane invaginates to generate intraluminal vesicles (ILVs). These ILVs are finally released into the extracellular space as exosomes, after the fusion of MVBs with the plasma membrane. On the other hand, ATG proteins such as ATG5, participate in exosome production (63). The location of LC3B on the lumen side of the ILVs suggests that a lipidation event takes place at the MVB membrane, or membrane invaginations. Besides, the fact that intact LC3B-positive EVs are eventually released, strongly suggests a secretory fate of selected autophagosomes by its fusion with MVBs in another vesicle called amphisomes (62). A recent article provided solid evidence regarding the involvement of SA in the EVs secretion. In this model, LC3-conjugation controlled the formation and secretion of EVs containing RNA-binding proteins (64). Nevertheless, the biological function of ATG5 and other ATG proteins in exosome production and release remains unclear (58). Finally, as it was recently reviewed, autophagy also participates in the control of conventional secretion, including selective types of autophagy such as such as ribophagy (65) and zymophagy (66, 67). It acts on the secretory apparatus at different steps, with selectivity between different secretory cell types (68).

## The Relevance of Secretory Autophagy in Degenerative, Endocrine, and Metabolic Disease

### Disease Linked With Aggregation-Prone Proteins Deposition and Accumulation May Be Associated With Disturbances in Secretory Autophagy

The potential implication of alterations in autophagy mechanisms in human health is the subject of strong research interest. Parkinson's disease (PD), Alzheimer's disease (AD), and another severe neurological diseases might be at least partially associated with alterations in degradative autophagy and/or in the autophagy-based secretion of certain peptides. This may be also the case for endocrine diseases. In neurodegenerative diseases such as PD and AD, as well as in endocrine and metabolic disease such as Diabetes mellitus, the SA mechanism may be altered in a way that toxic products are secreted and accumulated outside the cell as detritus that in time can cause cell death. In AD, Amyloid beta (Aβ) aggregates and accumulates outside the cell causing neuron functional impairment, structural changes and eventually cell death. The complete process reflects a severe imbalance between production, secretion, aggregation, and clearance of Aβ which progresses until the most advanced stages of the disease. Some studies have shown that autophagy plays a critical role in Aβ secretion (57, 69). Degradative autophagy blockade is followed by Aβ intracellular accumulation which has been demonstrated to be toxic to neurons as well as degradative autophagy in microglia is deteriorated by long exposure to Aβ (69). Moreover, the secretion of Aβ depends on autophagy, since Aβ secretion and plaque formation are reduced in mice lacking ATG7 in the excitatory forebrain neurons (57). It has been observed that ATG7 deficient mice tend to accumulate Aβ in the Golgi (57). Interestingly, it seems that autophagy machinery mediates the transport of certain peptides from the Golgi to endosomes (57). Alterations in these processes may affect Aβ secretion and promote the intracellular accumulation of Aβ, which in turn, results in toxic effects for cells. On the other hand, the administration of rapamycin, an autophagy activator, is followed by a reduction in the intracellular content of Aβ and an improvement in cognitive function in mice (57). Some alterations in SA have also been proposed as playing a role in the pathophysiology of the disease. Dysfunctional insulin-degrading enzyme (IDE), secretion may serve as an example of these SA alterations affecting Aβ aggregation (55). IDE -a ubiquitous enzyme of the inverzincin family of peptidases, is involved in the clearance of insulin, insulin-like growth factors, glucagon, amylin, and other peptides. IDE also degrades some neurotransmitters/neurohormones, including transforming growth factor-α, somatostatin, and endorphins. IDE inactivates calcitonin gene-related peptide and seems to modulate inflammatory responses, and the production of some tumor-associated antigens (70). Cellular and oxidative stress, insulin concentrations, free-fatty acid, and starvation seem to modulate IDE expression. IDE dysfunction may be associated with some forms of type 2 diabetes (T2DM), in humans and several single nucleotide polymorphisms in non-coding regions of the IDE gene, are associated with the disease (71–75). However, many IDE inhibitors have resulted in non-conclusive effects in terms of their potential as glucose tolerance improvers (76). As this enzyme is part of the catabolic pathways of insulin and insulin-like growth factors, high renal and hepatic expression of IDE is not a surprise under normal conditions. However, other cells such as astrocytes and some neurons also display relatively high levels of IDE expression (55). Predominantly located in the cytosol, IDE is also found in other organelles including endosomes, peroxisomes and mitochondria, and it is also found at the cell surface. A close to C terminus Sly sequence motif prevents IDE from enzymatic degradation when present at its lysosomal location (70). Usually <10% of the enzyme is secreted, following a non-conventional pathway. IDE secretion in microglia is enhanced by HMG-CoA reductase inhibitors (statins) (77). Having no signal sequence, IDE secretory pathway is mediated by autophagy. Son et al. (55) found that simvastatin is able to induce the degradation of extracellular Aβ40, which depended on IDE secretion from primary astrocytes. Simvastatin increased IDE secretion is mediated by the activation of autophagy through the LKB1-AMPK-mTOR signaling pathway in astrocytes. Importantly, IDE acts as a key protease for Aβ in the central nervous system. Also, Aβ induces IDE secretion involving the contribution of ATG genes. On the other hand, IDE secretion is associated with GORASP (Golgi reassembly and stacking protein) physiology (36). The dose dependent Aβ induced secretion of IDE is also mediated by GORASP and RAB8A. Moreover, the integrity of the autophagy flow is needed for this process. It also has been shown that Aβ injection in mice with alterations in ATG7 results in reduced expression and activity of IDE in the cerebrospinal fluid potentially associated with alterations in SA (55). Even when Aβ induced IDE secretion by astrocytes, it seems to fulfill many of the critical characteristics required to be considered an autophagy-mediated process. It is worth to be mentioned that some steps in this pathway are not completely understood. Interestingly, some epidemiological studies suggest that patients with T2DM are at a higher risk of developing AD. However, it is difficult to be certain about the nature of the link of both diseases with a dysfunctional SA. A standard oral agent for the treatment of type 2 diabetes (metformin), has shown some positive effects in experimental models of AD. Metformin decreases Beta-secretase 1 (BACE 1) activity which results in reduced production of Aβ (78). The inhibition of acetylcholinesterase (AChE) in the central nervous system, may explain at least in part, some beneficial effects on cognitive function, learning, and memory. Furthermore, Metformin reduces oxidative stress and exhibits anti-inflammatory properties (78). However, metformin is also an autophagy regulatory agent that may modulate both, degradative and secretory autophagy, and enhance autophagic clearance of intracellular neurofibrillary tangles formed by hyperphosphorylated tau protein (79). The real impact of these potential beneficial effects in clinical practice remains to be elucidated.

Amyloid Polypeptide and other aggregation-prone proteins accumulation surrounding the Langerhans Islet cells is a common finding in type 2 diabetes in humans. Islet Amyloid Polypeptide (IAPP) accumulation is long recognized as a phenomenon occurring in human type 2 diabetes. IAPP is co-synthetized in beta cells and secreted along with insulin, this is a 37-amino-acid residue polypeptide. Human IAPP forms oligomeric structures and fibrous extracellular precipitates that accumulate in the islets. The genetic background seems to be associated with the degree of IAPP deposition (80) and some nutritional factors (such as fat intake), would facilitate IAPP deposition (81). The implications of the IAPP accumulation in the evolution of human forms of diabetes is poorly understood. Furthermore, extracellular human IAPP can promote autophagy in beta cells and the reactive Oxygen Species (ROS), mediates part of the IAPP induced degradative autophagy by involving the AMPK pathway (82). On the other hand, it has been suggested that intracellular IAPP oligomer formation would be toxic for beta cells. In this way, IAPP oligomers may damage mitochondrial membranes as well as the endoplasmic reticulum (ER) (83). In humans, the augmented expression of IAPP in beta cells is followed by an increase of the autophagy flux (84). Moreover, lack of autophagy in hIAPP-expressing animals resulted in hIAPP oligomer and amyloid accumulation in pancreatic islets, leading to β cell death (83, 85–87). It has been suggested that IAPP oligomers may cross plasma membranes inducing damage by a “prion-like” effect. In this way, the intraperitoneal injection of IAPP aggregates from the pancreas homogenate to the transgenic mouse that expresses hIAPP, dramatically accelerates IAPP amyloid deposition, which was accompanied by abnormalities resembling T2DM (88, 89). In this process, degradative autophagy integrity seems to be of relevance. In general, increased degradative autophagy would protect against IAPP induced damage on beta cells. However, evidence on the potential role of SA remains less conclusive. Alterations in IDE secretion has been suggested as part of the pathophysiology of IAPP deposition. IAPP is a substrate of IDE. However, IDE inhibition does not seem to increase amyloid deposition of endogenous IAPP *in vivo* (90). The recently described expression of another aggregable protein, α-synuclein, in beta cells adds complexity to the landscape of aggregation-prone proteins induced damage in type 2 diabetes. Alpha-synuclein expresses in murine pancreatic islets and exogenous overexpression of α-synuclein reduces insulin secretion by INS1 cells (91). It has been proposed that α-synuclein interacts with the Kir6.2 subunit at the KATP channel located at the beta-cell membrane suppressing insulin secretion (91). In normal conditions, this mechanism may protect beta-cell from ER stress by down-regulating exaggerated insulin secretion. In the presence of type 2 diabetes, α-synuclein may play a negative role in insulin release contributing to hyperglycemia, oxidative stress, and glycosylation of protein (91). Steneberg et al. (56) showed that autophagic flux is reduced by increased levels of α-synuclein present in β-cells from IDE KO mice and in T2D patients. On the other hand, Sharma et al. (92), proposed that α-synuclein is able to activate IDE, while IDE inhibits amyloid formation by α-synuclein. However, it is unclear if these factors may result in increased IAPP deposition in T2DM in humans. Moreover, several reported changes may represent indirect evidence of the IAPP induced damage and cell adaptation. Also, these alterations might precede or follow the intracellular accumulation of oligomers. Finally, direct or indirect alterations in SA may result in an increased concentration of proinflammatory mediators such as IL-1β increasing IAPP deposition, functional alteration, oxidative stress, and cell death.

In PD's Disease (PD), the accumulation of α-synuclein, a protein with high binding affinity for smaller vesicles (as synaptic vesicles, for instance), was reported. Alpha-synuclein mutation or over-expression impairs membrane trafficking including exocytosis and ER-to-Golgi transport (93, 94). These alterations are associated with ER stress, increasing oxidative stress affecting cell homeostasis. Moreover, lysosome impairment seems to play a critical role in the progression of the disease (95). It has been proposed that the Secretory Carrier Membrane Protein 5 (SCAMP5), promotes the secretion of α-synuclein and other neurotoxic proteins via exosomes (50). It has been demonstrated that SCAMP5 has some effects as an inhibitor of degradative autophagy, playing role in balancing several processes involved in cell homeostasis, including vesicle trafficking as well as constitutive, degradative and secretory autophagy (50). Also, several other alterations linked to autophagy have been identified in different models of PD. Alterations in the TMEM230 gene (Transmembrane Protein 230), have been recently associated with the pathophysiology of familial forms of the disease. TMEM230 regulates the autophagy-mediated clearance of α-synuclein and mediates Rab8a-associated SA. As a result, this protein regulates Golgi-derived vesicle secretion (96). Lewy-bodies formation, which is typically associated with cognitive dysfunction and dementia seen in some patients with advanced PD, may result from these alterations (96). PARK7/DJ-1 protein has been associated with PD and certain forms of cancer. PARK7/DJ-1 participates in the regulation of several cell processes, including anti-oxidative protection. Furthermore, an increased pro-oxidative environment is associated with degenerative damage along with the disease evolution. The autophagy pathway involving ATG molecules was associated with PARK7/DJ-1 secretion (51). Thus, impairment in autophagy might result in the defective secretion of this protein with potential implications in terms of anti-oxidative stress protection, playing a role in PD progression.

Amyotrophic lateral sclerosis (ALS), is a degenerative disorder that affects cortical, bulbar and spinal motor neurons and is mainly characterized by a progressive adult-onset. Like other neurodegenerative diseases, ALS is categorized as a “proteinopathy” since SOD1, TDP-43, and FUS are pathological proteins that accumulate, interfere, and impair neuronal functions leading to cell death. Sproviero et al. suggest that these toxic proteins are transported mainly by EVs that might play a role in prion-like propagation of ALS disease (97).

### Impairment in SA May Affect the Effect and the Concentration of Secreted Peptides and Other Co-secreted Substances

Disruptions in SA may result in relevant changes in the concentrations of secreted peptides and other co-secreted molecules, as well as in their paracrine and endocrine effects.

Altered secretion of lysozyme is related to some forms of inflammatory bowel disease and relevant changes in host defense mechanism. Paneth cells are specialized secretory cells in the small intestine that have been related to Crohn disease through a mutation of ATG16L1 (98). It has been observed, that lysozyme and other critical secreted antibacterial proteins are rerouted by Paneth cells through SA as a reaction to bacterial invasion. Moreover, activation of innate lymphoid cells type 3 (ILC3) which secrete IL22 licenses Paneth cells to secrete lysozyme through SA (52, 99). These data suggest that Crohn disease may be associated, at least in part, to some genetically mediated alterations in SA.

Impaired secretion of other inflammation regulatory molecules by a disrupted SA has been reported. IL-1β is a major pro-inflammatory cytokine, that also doesn't follow the classical endoplasmic reticulum-to-Golgi route. IL-1β release is mediated by SA requiring the participation of the microtubule-associated protein EB1 (100). This autophagy-dependent, unconventional secretion pathway is of special interest (45). IL-1β transcription is induced by different stimuli ranging from microbiological agents to other cytokines and also growth factors (101). Moreover, neutrophils, macrophages, and microglia are relevant sources of IL-1β under infectious and other conditions and it was demonstrated that autophagy is responsible for IL-1β exocytosis under these challenges. As expected for autophagy-based secretory mechanisms, it was shown that siRNA-mediated knockdown of ATG5 reduces IL-1β secretion in neutrophils (54). In contrast, cell starvation increases the colocalization of IL-1β and LC3B promoting IL-1β secretion. Extending data including all data mentioned above strongly suggests that SA is involved in IL-1β secretion by neutrophils and other secreting cells.

IL-1β derives from an inactive precursor, pro- IL-1β, that requires cleavage by caspase-1 for activation. Activation of caspase 1 is mediated by the protein complex called ‘inflammasome’. Release of mature IL-1β relays on SA and it has been suggested that GRASP (a Golgi apparatus associated factor) participates in IL-1β secretion (45). After release, a series of inhibitory molecules including IL-1Ra, sIL-1RI, sIL-1RII, and sIL-1RAcP regulates IL-1β mediated inflammation. IL-1β secretion has been demonstrated to be increased in several endocrine, metabolic and degenerative conditions, as well as some acute entities and infections. In fact, a key role in the defense against microbial pathogens and in tissue injury repair, is performed by IL-1β. Local and systemic responses to this cytokine are responsible for homeostatic effects. Exaggerated responses to IL-1β, however, are associated with potentially deleterious effects; and excessive IL-1β activity is associated to vasculitis and thrombosis. IL-1β also plays a role in rheumatoid arthritis and other inflammatory diseases. Part of the structural damage associated with PD, AD, and other degenerative entities seems to be linked to IL-1β effects. For instance, β cells exposure to increased concentrations of IL-1β is associated with functional deterioration and cell death. Moreover, IL-1β is linked to inflammation in obesity, type 2 diabetes, insulin-resistance associated entities (e.g., polycystic ovary syndrome), atherosclerosis and other conditions. In addition, IL-1β seems to play a role in the pathogenesis of Autoimmune Thyroid Diseases (102). Regarding type 2 diabetes, only when basal levels of IL-1β mRNA are low, hyperglycemia induced IL-1β production in β cells can be observed (103). IL-1β is also produced by infiltrating immune cells in the pancreas (104). Taking all this information into account, it seems that IL-1β acts as a “metabolic sensor” aside of its well-recognized role as pro-inflammatory mediator (105). IL-1β physiology is a highly illustrative example of the complexities of degradative and secretory autophagic processes. Hence, the induction of inflammasomes triggers autophagosome formation in macrophages (106). In some tissues, this effect may be part of a negative mechanism to control and limit the inflammatory response confronting challenges of infectious origin. It seems to be also linked to an increased cytokine-mediated anti-microbial defense.

In some cases, IL-1β was found to be increased in degradative autophagy and may be linked to tissue damage. In pancreatic acinar cells, IL-1β hyperactivity seems to be associated with increased endoplasmic reticulum stress-inducing autophagy. This mechanism may be related to an impaired autophagic flux leading to trypsin activation and pancreatic injury. It has been observed that Atg 5 alterations are followed by an increment in IL-1β plasma concentration though pro-IL-1β caspase-mediated cleavage (107). As mentioned before, autophagy is critical to IL-1β release through the SA mechanism that involves the AIM2 inflammasome. The interplay between different cytokines (many of them affecting autophagy in several manners), adds complexity to this homeostatic network. This effect may result in a chain of autophagic process modifications affecting the SA of regulatory peptides. Although precise mechanisms articulating the balance between the complex processes enumerated above remain unclear, many of them seem to be of major relevance for a better understanding of the immune-inflammatory components in degenerative and metabolic conditions.

Autoimmune thyroiditis (AIT) is among the main causes of hypothyroidism in human. An increased expression of some inflammasome components as well as IL-1β was observed in thyroid gland tissues from AIT patients (108). This phenomenon suggests an upregulated SA activity in these subjects and may play a role in the pathophysiology of the disease. Other autoimmune endocrinopathies may also reflect a certain degree of dysfunctional SA with dysregulated secretion of IL-1β and other cytokines. Elevated levels of pro-osteoporotic cytokines including IL-1β have been found in patients with different forms of hyperthyroidism (109). IL-1β secreted by peripheral monocytes induces IL-6 secretion by stromal cells and osteoblast, IL-6 increments osteoclast proliferation and differentiation inducing increased bone resorption (110) A role of IL-1β hypersecretion cannot be excluded as a potential mediator of hyperthyroidism associated altered bone resorption (111).

Emerging role of secretory autophagy in carcinogenesis and endocrine tumor progression is a topic of significant clinical relevance. Secretory autophagy may be involved in the secretion of tumor-promoting proteins. It has been suggested, that low expression of Rasal2 gene was associated with the recurrence of luminal B breast cancer (112). Recently has been shown that the Rasal2 gene knock out induces secretory autophagy. Although the potential mediators are not elucidated, the increase in SA seems to be associated with luminal breast cancer proliferation (113). Altered secretory patterns of IL-1β, IL-6, IL-8, bFGF, and other growth factors have been demonstrated for certain tumor types (114). Interestingly, IDE is expressed in some human undifferentiated breast and ovarian types of cancer, as well as in retinoblastoma. A tumor-suppressing activity was suggested for this enzyme (115, 116). Dysfunctional SA of IDE might play a role in very aggressive tumors. Paracrine secretion between cancer-cell interactions would facilitate tumor initiation, growth, and spreading. It has been suggested that paracrine secretion of IL6, IL8, and bFGF induces autophagy in head and neck cancer-associated fibroblasts (114). Therefore, SA and degradative autophagy modulates critical interactions between tumor cells and the surrounding microenvironment and may result in significant metabolic modifications, with a strong impact in cancer cell adaptations by Warburg and reverse Warburg effects (117).

## Conclusions

Macroautophagy is a complex cellular pathway characterized by the formation of the double-membrane vesicle called autophagosome that sequesters cytoplasmic contents to be delivered to the lysosomal compartment for degradation. However, increased experimental evidences strongly support that another fate of the autophagosome biogenesis molecular machinery exists, leading to a novel pathway of unconventional secretion. This last process was named SA to differentiate it from the canonical degradative autophagy (6, 61).

SA has been recognized as a novel mechanism to explain the secretion of a series of peptides which have critical biological importance. Although the molecular pathways and vesicular trafficking in SA are not fully elucidated, canonical autophagy machinery is mechanistically involved (7). Thus, the autophagy-related proteins, such as ATG5 and several other components of the autophagosome biogenesis (13) play a central role in the secretion of critical disease-related proteins. Indeed, autophagosome formation seems to be involved in the eventual release of these relevant proteins (8). Interestingly, the fact that cargo recognition molecules, such as the TRIM family are mechanistically involved in this process (7) strongly suggests that SA could be considered as a non-canonical type of selective autophagy. In SA the process does not end in lysosomal degradation but in the secretion of the selected cargo. A wide range of proteins secreted by mammalian cells following different pathways are related to autophagy and associated with human diseases. This includes proteins related to some endocrine and aging associated diseases.

Degradative and secretory autophagy are two integrated multistep processes highly regulated by several physiological and disease-related factors. Starvation, oxidative stress, and hypoxia are well recognized stimuli for degradative autophagy. SA efficiency depends at least in part, on the integrity of the molecular machinery involved in the degradative processes. Changes in degradative and secretory autophagy may result in impairment of the release of different peptides that, in turn, may affect autophagy in other cells by paracrine or even endocrine ways. Implications of this interplay in the prevention, prognosis and treatment of several diseases are still to be elucidated.

Multiple neurodegenerative disorders have in common abnormal protein accumulation and aggregation (32). Autophagosomes or MVBs would be able to sequester and degrade cytosolic-protein aggregates through lysosomes. Externalization of protein aggregates may also be mediated by secretory autophagy. On the other hand, changes in the secretion of protein aggregates might decrease the proteotoxic stress in the releasing cells or reduce the spreading of protein aggregates to neighboring cells.

There is substantial experimental data that allow us to consider that canonical degradative autophagy and its related factors are mechanistically associated with secretion. It has been demonstrated that SA requires the autophagic pathway and both, secretory and degradative autophagy are integrated and highly regulated processes that interact in ultimate cross-talking molecular mechanisms. Impaired secretory autophagy may result in the aggregation-prone proteins deposition and accumulation, or severe alterations in the release and concentration of other secreted proteins. The relevance and the mechanisms involved in these interactions seem to be very important in metabolic and degenerative diseases. The pharmacological modulation of SA and its regulatory pathways might also be a clear target for drug research. Although there is available evidence that outline the potential relevance of the pharmacological control of SA, important gaps in the evidence remain to be filled. Metformin, for instance, may modulate SA by its demonstrated effects on degradative autophagy. It has been observed that in patients with type 2 diabetes, metformin upregulates mitophagy, and improves mitochondrial function in a glucose-lowering independent manner (118). By inhibiting macrophage activation and activating autophagic flux, metformin reduces pro-inflammatory cytokines release (including IL-1β) (119). However, a direct effect on SA mechanisms cannot be excluded. Statins (HMGCoA reductase inhibitors) increases IDE release by astrocytes in a dose-dependent manner. These agents directly modulate SA at least in some tissues. However, the potential relevance of this mechanism on the beneficial and/or adverse events associated with statin use in practice remains obscure. Iron enhances Aβ stimulated IL-1β secretion in microglia (120). The impact of this finding in humans is still unknown. Many other agents seem to modulate IL-1β secretion by regulating SA processes in different ways, and in different cell types (121). Translation of these findings to drug development and application into clinical practice will take a while. However, the potential implication of impairment in secretory autophagy, its cargos, pathways, and regulation in human diseases such as metabolic/aging pathological processes is a clear focus of biomedical investigation. Further research on secretory autophagy pathways as a potential target of therapeutic intervention is deserved.

## Author Contributions

MV selected the subject, wrote the 1st and 2nd sections, and the conclusions, selected part of the bibliography, reviewed the whole manuscript, and made the table. CG wrote the abstract and the third part of the manuscript and selected part of the bibliography and reviewed the manuscript. RR collaborated in the writing of the whole manuscript and made the figures.

## Conflict of Interest

The authors declare that the research was conducted in the absence of any commercial or financial relationships that could be construed as a potential conflict of interest.
